# Future Green Technology: A Freezing Water Micro-Droplet as an Optical Switch Based on a Time-Domain Photonic Hook

**DOI:** 10.3390/nano13152168

**Published:** 2023-07-26

**Authors:** Oleg V. Minin, Yinghui Cao, Igor V. Minin

**Affiliations:** 1 Nondestructive Testing School, Tomsk Polytechnic University, 36 Lenin Avenue, Tomsk 634050, Russia; ovminin@tpu.ru; 2 College of Computer Science and Technology, Jilin University, 2699 Qianjin Street, Changchun 130012, China; caoyh@jlu.edu.cn

**Keywords:** water droplet, freezing, photonic hook, mesotronics, optical switch

## Abstract

This paper pays attention to the broader interest of freezing water droplets in mesotronics, particularly to their use as a new all-optical device platform. Here, we show that a freezing mesoscale water droplet with a low Bond number can behave as fully biocompatible natural microlense to form a photonic hook for application in a tunable temperature-controlled optical switch. We first introduced and demonstrated the basic concepts of an optical switch without changes in the wavelength of illumination of a particle or any moving parts being involved. The principle of the operation of the switch is based on the temperature-induced phase change inside the water droplet’s refractive index. The simulation results show that the optical isolation of switched channels for an optical switch with linear dimensions of about 15 λ^3^ based on a freezing water droplet can reach 10 dB in the process of temperature variation at a fixed wavelength. The use of freezing mesoscale droplets acting as a time-domain photonic hook generator open an intriguing route for optical switching in multifunctional green electronics tools for sensing, integrated optics and optical computers.

## 1. Introduction

The field of optical switching has a long history and is too broad [1]. Several technologies have been studied for use as spatial- or wavelength-selective optical switches [2]. A critical comparison of various types of optical switches was analyzed in [3]. Instead of using a wavelength-selective optical switch, it is possible to use the switching spatial orientation of the recently discovered (2015) photonic hook phenomenon, which makes it possible to numerically [4,5] and experimentally [6] demonstrate the concept of a new all-optical switch.

At the same time, natural materials such as water have shown potential for use in future green technologies [7,8,9]. Using a single water droplet as a photonic component with different optical properties has become a new intriguing paradigm in mesotronics (mesoscale photonics) for wavefront manipulation and unusual applications [10,11,12,13,14,15].

Phase-change materials such as a freezing water droplet are suitable for fulfilling optical switching functions due to their temperature sensitivity and relatively fast switching dynamics. Geometrically symmetrical (spherical) water droplets with refractive index asymmetry due to freezing are used to obtain a curved photon flux. Such freezing droplets are the so-called Janus particles obtained by combining two materials (water and ice in our case) with different optical properties [16].

Recently, we demonstrated the possibility of generating a self-bending photonic hook in the time domain (TD-PH) based on a freezing water droplet [17]. A key property of a photonic hook for realizing the function of an optical switch is the dependence of the curvature of its beam on the position of the water–ice interface in the process of the droplet freezing. Therefore, with a certain dynamically changing internal structure of a freezing droplet and reception zones, it is possible to achieve a change in the magnitude of the optical signal in each of the channels when the water phase changes in the process of freezing. An optical switch based on the photonic hook effect is a so-called forward-scattering device. Thus, its optical attenuation is sensitive to the spatial position and the distance between the droplet and the detectors. Thus, photonic hooks could function like switching channels after passing freezing-droplet Janus particles. Such freezing-water-droplet-based optical switches are very attractive due their potentiality for integration and their wavelength-scaled small size.

## 2. Model

As shown in [17], the formation of a localized curved beam in the form of a TD-PH by freezing a mesoscale water droplet immersed in air (*n* = 1) was studied by using the Comsol Multiphysics commercial software (Ver. 5.3) based on the finite-elements method. A few words about the shape of the droplet are given in [18]. The Bond number (Bo) characterizes the effects of the surface tension and gravity:

$$Bo=\frac{\Delta \rho gR^{2}}{\sigma },$$

where σ is the surface tension of the droplet, Δ*ρ* is the density difference between the droplet and the surrounding medium, g is a gravitational constant and R is the radius of the droplet.

It should be noted that the contact angle is not included into the Bond number, Bo (which is more than 150 degrees for super-hydrophobic surfaces), so Bo is unable to characterize the effects of interactions between solids and liquids. The minimum droplet radius on the surface decreases with decrease in the Bond number, but the behavior of the minimum droplet radius with the Bond number is not linear due to the energy balance [19]. In micron-sized water droplets, the Laplacian (capillary) pressure is much greater than the hydrostatic pressure (due to gravity). In this case, we can write:

$$\frac{\sigma }{R}>>\rho gR,\;\mathrm{thus}\;R<<\sqrt{\frac{\sigma }{\rho g}}=\sqrt{\frac{72\times 10^{-3}}{10^{3}\times 9.8}}\approx 2.7\mathrm{mm}.$$

So, for micron-sized droplets, the shape of a droplet with a small Bond number may be considered as almost spherical [17,20], which has a minimum surface area for a fixed volume.

The process of droplet solidification and freezing is dependent on the following key factors: physical, dynamic and thermal. An experimental study on the dynamics of the evolution of the water–ice interface upon freezing was discussed, for example, in [21]. In addition, the shape of a water droplet deposited on a cooled surface cannot be an ideal sphere in some cases [21]. It was shown that when the interface moves up above the center, its curvature changes and it is no longer a spherical curve. Importantly, the experiment of water drop freezing in [21] was performed in a Hele–Shaw cell apparatus, which consists of two parallel flat plates separated by a small gap, in between which the droplet is freezing. Although the experimental studies discussed above were related to millimeter-scale droplets, the experimental conditions corresponded to a two-dimensional model. Therefore, in contrast to [17], we introduced a small distortion of the shape of a spherical droplet and the shape of the water–ice interface according to the results of [21] proportional to the droplet radius. To mimic the experimentally observed freezing water drops in a Hele–Shaw cell [21], the boundary of the water–ice droplet model used in this study consisted of Bezier curve segments, with its sizes along the x and y axes being D_x_ = 5.25 µm and D_y_ = 5 µm, respectively. Furthermore, the water–ice interface front of the freezing water droplet model consisted of a circular arc with different curvatures.

**Figure 1:**
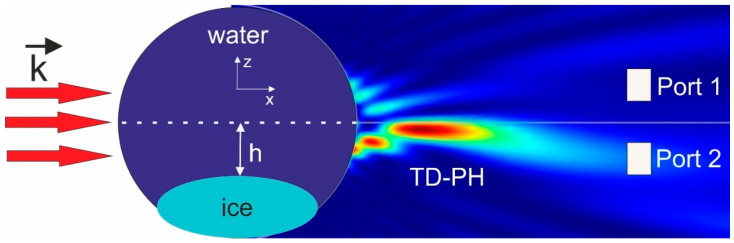
A schematic diagram of the time-domain photonic hook formation by freezing water droplet for optical switch. Two-channel receivers, Port 1 and Port 2, are schematically shown as white squares, and h is the position of the ice–water interface.

As in [17], and taking into account [22], we used a 2D geometry in the simulations to reduce the computational time. A perfectly matched layer (PML) was applied for the simulation of the boundary conditions. Outside and inside the droplet, the mesh size was λ/8 and λ/15, respectively. The total number of elements of the discretized computational domain was about 7 × 10^4^, and the number of degrees of freedom solved was about 1.5 × 10^5^. The incident plane wave propagating along the *x*-axis was linearly polarized along the *y*-axis. It was assumed that ice and water are materials without any inhomogeneities and inclusions. The refractive indices of ice and water at the wavelength of λ = 589 nm are 1.201 and 1.334, respectively [23]. A schematic diagram of the optical switch based on the time-domain photonic hook formation by freezing a water droplet is shown in Figure 1. One can see that the optical switch had one input port and at least two optical output ports.

It should be noted that for the net optical isolation of Port 1 and Port 2, it was preferable to set the input aperture mask of these receiving ports according to the cross-section of the photonic hook in the plain of the receivers. It is known that due to the wavelength-scaled dimensions of the water droplet, the characteristic diameter of the photonic hook measured at the deflection point corresponding to the point of maximum intensity along the photonic hook was in the order of the illuminating wavelength, and the size of the photonic hook’s beam waist broadened along the photonic hook due to the diffraction. In our simulations, the diameter of the receiving port apertures were set to about 1 μm.

## 3. Results and Discussion

**Figure 2:**
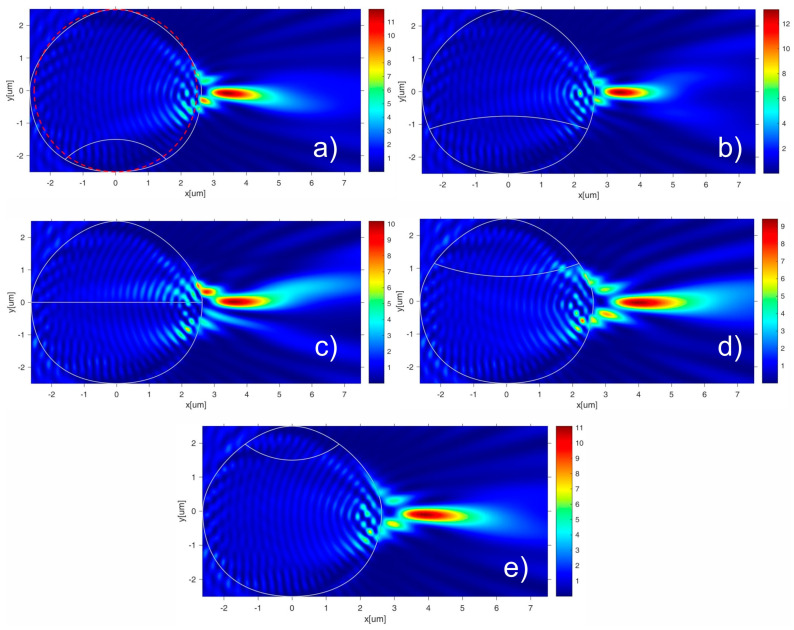
The evolution of the photonic hook’s shape and water–ice interface in the process of freezing the water droplet. Red circle shows the spherical droplet shape, and white counter corresponds to the distortion of the droplet shape with diameters of D_x_ = 5.25 µm and D_y_ = 5 µm. White curve inside the droplet shows the shape and position, h, of the water–ice interface. The electric field intensity enhancement (E/E_0_)^2^ is shown as follows: (**a**) h = −1.5 µm, R_in_ = 0.9R, (**b**) h = −0.75 µm, R_in_ = 3R, (**c**) h = 0, (**d**) h = 0.75 µm, R_in_ = 2.5R and (**e**) h = 1.5 µm, R_in_ = 0.9R.

The evolution of the photonic hook’s shape and the water–ice interface (h, R_in_) during the freezing of a water droplet is shown in Figure 2 for a droplet with a radius of R = 2.5 microns. Such a scenario of water–ice interface propagation at different times corresponded to the model discussed in [20,21].

One can see that at the early stage of the freezing, the water–ice interface front had a convex shape; at the last stages the curvature of the water–ice interface front, it is inverted towards a concave geometry, as show in Figure 2a–e. The geometric shape of the water–ice drop model as well as its freezing front were very similar to the experimentally observed results in [21]. In general, the dynamics of the change and formation of the photonic hook coincided with the previously considered properties of a spherical water droplet [17]. The unique properties of the TD-PH involved the changing of the angle of its bending and curvature with respect to the temperature of the droplet and maintained a high spatial localization at distances greater than the Rayleigh diffraction length [24,25]. One can see (Figure 2) that the curvature and the shape of the water–ice interface changed with the different position, h, of the interface. It can also be clearly seen that the propagation path of the localized field at the shadow side of the droplet was deflected at the inflection point near the position of I_max_ = max(|E/E_0_|^2^), resulting in the bending of TD-PH. Note that the water–ice interface was proportional to the square root of the freezing time [20].

Due to the asymmetry of the freezing water–ice droplet, the bottom half of the droplet contained less ice than the top half at the initial stage; thus, the photonic hook was bent downward toward the bottom of the droplet when h = −1.5 µm (Figure 2a). For h = 0 (Figure 2c), the top half of the droplet was water and the bottom half of the droplet was ice, so the top half of the droplet was optically thicker than the bottom one due to the differences in their refractive indices, and the photonic hook was bent upward. For large values of h, the situation with the orientation of the photonic hook was reversed, and the TD-PH was bent down again (Figure 2e).

Such dynamics of changing the orientation of the photonic hook during the water droplet freezing process made it possible to use the considered effect to create an optical switch.

**Figure 3:**
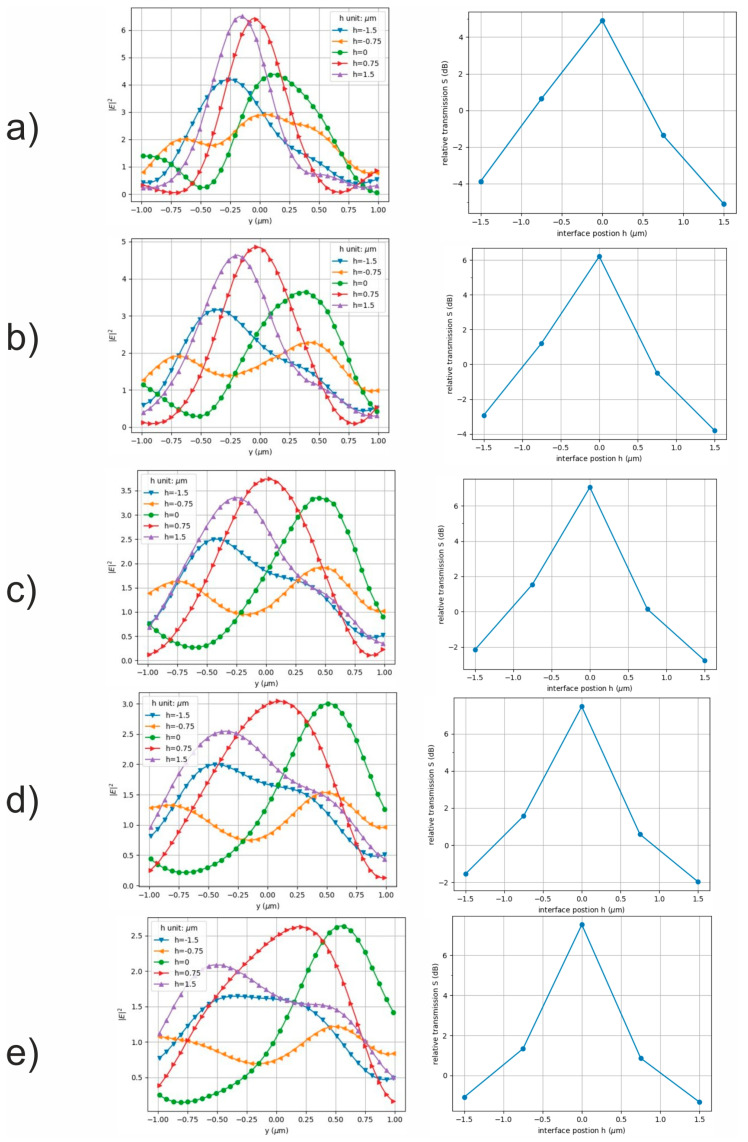
Electric field intensity distributions along *y*-axis (left column) and relative transmission, S (right column), for different positions of Port 1 and Port 2 lines along *x*-axis: (**a**) x = 5.0 µm, (**b**) x = 5.5 µm, (**c**) x = 6.0 µm, (**d**) x = 6.5 µm and (**e**) x = 7.0 µm.

The electric field intensity distributions along the *y*-axis and the relative energy transmission, *S*, for the two-channel receivers, calculated as S = 10 × log(W_1_/W_2_) at Port 1 (0.1 µm < y < 0.7 µm) and Port 2 (−0.7 µm < y < 0.1 µm) (see Figure 1) for the different positions of the Port 1 and Port 2 planes along the *x*-axis, are shown in Figure 3.

Following [4], the dependence of the relative energy transmission *S =* log(*W*_1_/*W*_2_) on the position of the water–ice interface inside the droplet, where the energies, *W*_1,2_, are the integral of the electric field intensity over the cross-sectional area, Σ_1,2_, of the corresponding receiving port (1 or 2), is as follows:

$$W_{1,2}=\int_{\Sigma_{1,2}}|E{|}^{2}d\sigma .$$

The extremes on these curves corresponded to the alternative states of the optical switch, when either the signal S_2_ from the second port, Port 2, (see Figure 1) or the signal S_1_ from the first port, Port 1, prevailed. In Figure 1, such states are marked with large white squares.

One can see from Figure 3 that when the position of the water–ice interface boundary (h) changed, the field intensity maximum in the plane of Ports 1 and 2 changed its position. This trend was the same for the different observation planes along the *x*-axis, but the law of the change in the position of the field intensity maximum was somewhat different. Accordingly, the maximum value of the relative energy transmission also changed.

**Figure 4:**
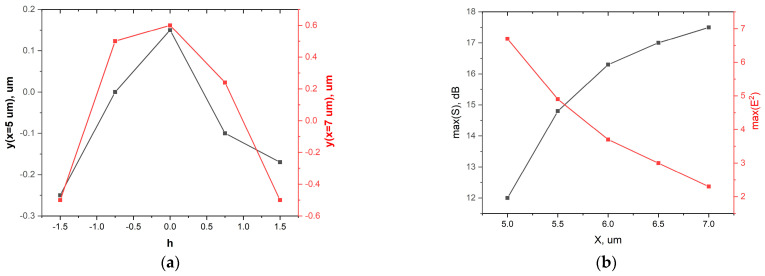
The position of maximal field intensity along the *y*-axis for x = 5 µm and x = 7 µm vs. water–ice interface position h (**a**); maximum of the relative energy transmission, *S*, and maximal field intensity enhancement vs. position of the port planes along *x*-axis (**b**).

**Figure 5:**
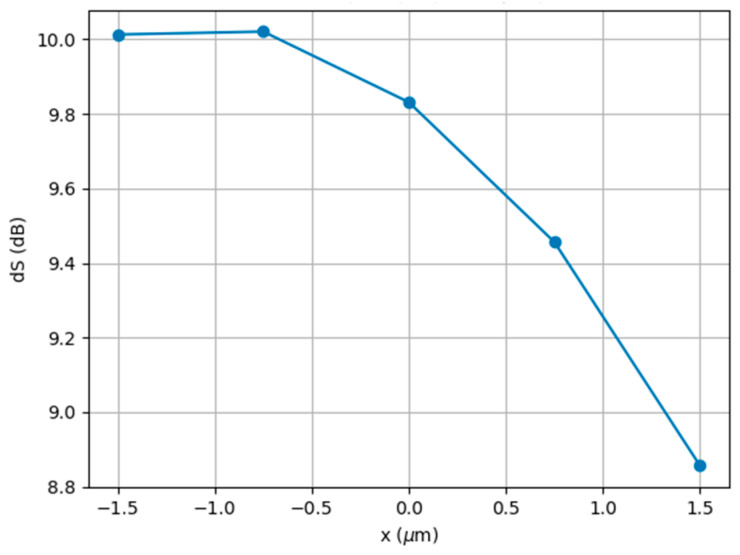
Relative energy transmission *dS* = *S*_*h*=0_ − *S*_*h*=1.5__µm_ of receiving ports versus the position of the port planes along the *x*-axis.

This statement was confirmed by Figure 4, which shows the position of the field intensity maximum in the plane of Ports 1–2 for various distances from the shadow surface of the droplet (a) and the dependence of the maximum of the relative energy transmission, S, and the maximum field intensity on the position of the observation plane (b). It is quite obvious that when the observation plane (the position of the ports along the *x*-axis) moved away from the shadow surface of the droplet, the value of the maximum relative energy transmission increased with the decrease in the maximum value of the field intensity enhancement. At the same time, the signal difference, *dS* = *S*_1_ − *S*_2_, could serve as a measure of the optical decoupling (isolation) of the switching channels [4]. It is noteworthy that, under the condition of S ≈ 0, the optical switch under discussion could be considered as an optical splitter, since the optical energies in Port 1 and Port 2 tended to be equal. In this case, the splitting losses could be substantial due to the open nature of the proposed types of optical switching [4]. One can see (Figure 5) that the maximum relative energy transmission, *dS*, could reach a value of 10 dB under the considered conditions. We emphasize that we did not optimize the optical switch, but we only demonstrated a possible physical concept. Larger S values can be achieved by using other droplet diameters or materials when the refractive index contrast is higher than that considered in this study.

For an optical switch, a minimal transition time from one switch state to another is strongly desired. As for the operating speed of the optical switch based on a freezing droplet, a one-dimensional analytical solution of a millimeter-scale freezing spherical water droplet moving in cold air was discussed in [26] in the case of a uniform temperature distribution over the liquid’s core [27]. A three-dimensional simulation showed the fast freezing dynamics of micro-sized water droplets with radii of 5 μm and 0.5 μm [28]. With the decrease in the diameter of a water drop, multiple nucleation centers are formed, which accelerate the freezing of the drop. According to [29], for a hemispherical droplet, the freezing time, *t*, can be expressed as $t\sim A\left(\frac{3V}{\pi }\right){\left(\frac{1-\cos\left(\theta \right)}{2+\cos\left(\theta \right)}\right)}^{2/3}\frac{1}{T}$, or for a droplet on a superhydrophobic surface (with a contact angle θ = 180 deg.), $t\sim 1.58A\left(\frac{3V}{\pi }\right)\frac{1}{T}$, where *T* is the cold surface temperature, *A* is a constant determined by the droplet itself and *V* is the initial droplet volume. For a 50 μm droplet, the experimental freezing time is of the order of 1 ms [30]. In addition, experiments have shown that the recalescence time for water droplets with D~20 μm are approximately 5 ms and 5 μs for air temperatures of −4 °C and −20 °C, respectively [31]. Numerical simulations of the freezing dynamics of supercooled water droplets with 10 μm and 1 μm diameters have shown [28] that the freezing time is independent of the number of nucleation sites. The average propagating velocity of the ice–water interface for a 1 μm droplet is about 0.036 m/s, and it is about 0.028 m/s for a 10 μm droplet. Therefore, a 10 μm water droplet completes its freezing at around 2.4 ms, depending on the freezing scenario [28]. The switching speed is reasonably fast. Devices based on such a concept have potential applications in optical attenuators and, probably, in displays and other applications where millisecond-scale switching times are acceptable. This time is comparable, for example, with liquid-crystal-based optical switches [32]. The decay time and rise time for an optical switch using a deformable liquid droplet with a size of about ~140 μm (an order of magnitude larger than the concept under consideration) and covered by a ~30 μm black liquid layer are ~32 ms and ~18 ms, respectively [33]. An Agilent optical switch uses bubbles in a fluid heated by an attached silicon substrate, where the optical transmission decreases to zero in a time of ~20 ms [34]. Thermo-optical switching speeds depend on the switch design. When a thermo-optical switch is based on the use of a high-thermal-conductivity material, a short switching time is achieved at high power and vice versa; a low switching power is available when using a low-thermal-conductivity material, but the switching time is long. Thermo-optical switching speeds range from 100 µm to several milliseconds [35].

To create a heat flux for freezing/unfreezing, the thermoelectric Peltier effect [36] can be used in supporting plates, i.e., a water droplet may be placed on Peltier element with a superhydrophobic surface [37,38]. One Peltier element face can be heated while the opposite face is simultaneously cooled. It is important to note that this effect may be reversed, whereby a change in the applied DC voltage polarity will cause heat to move in the opposite direction [39]. Consequently, a thermoelectric Peltier element may be used for both heating and cooling, thereby making it highly suitable for freezing and unfreezing water droplets. It is possible to reduce the switching time by increasing the thermal isolation between the surrounding medium and the water droplet. All structures (the Peltier element, droplet and detectors) may be placed on a thermoelectric module, similar to [40].

## 4. Conclusions

Many different optical switching concepts are under development. Understanding and developing a variety of methods and mechanisms of optical switching is important to implement their required functions. The concept of water-based mesoscale photonics, where freezing a water droplet could be a new platform for novel systems and devices, was introduced in this paper. It suggests that the solid and liquid states of a water droplet can allow for the ready shaping and exploitation of many optical effects, including optical switching, in ways not previously considered. We successfully demonstrated a new method of generating mesoscale freezing droplets that can work as a flexible and temperature-tunable optical switch. The switching effect is realized by changing the photonic hook space configuration in the transmission mode by freezing a Janus water droplet with temperature variation. Such a switch can be attributed to the class of a temperature-controlled type [41,42] that reversibly convert between the off/on states by external thermal stimuli (such as cold or heat). The phenomenon of a TD-PH is the key to the efficient manipulation of light at the mesoscale based on natural materials. We showed the fundamental possibility of developing an optical mesoscale (with a size of the order of the wavelength) two-channel switch based on a freezing water droplet. Due to the unique properties of the time-domain photonic hook to change the curvature with respect to the temperature, this switch is a potential candidate for the implementation of optical switching in future miniature ‘on-a-chip’ devices and green optoelectronics and mesotronics [43] without the use of any micromechanical devices and in the absence of electrical signal control or multi-wavelength illumination.

It should be noted that larger *S* values can be achieved by taking into account the fact that in a freezing droplet, the ice may be a porous medium rather than completely solid ice due to bubble formation inside the droplet [44], and the refractive index contrast may be higher than that considered. Moreover, since water as a phase-change material is fluid in its liquid state, it is advisable to use microencapsulation methods to ensure the reusability of the switch. Microencapsulation makes it possible to obtain a material with a phase change by enclosing it in capsules with sizes of less than one micron [45,46]. Switches of this type are cheap and simple compared, for example, with metasurface-based temperature-controlled optical switches [47].

It should be noted that in this work, only a proof-of-concept for an optical miniature water-based time-domain photonic hook switching is presented, and we did not carry out a full-scale optimization of the full optical switch characteristics. The speed of the optical switch based on a freezing water droplet was sufficient for routing applications and for circuit switching. The drawbacks include heat power dissipation. The authors hope that a future optical computer that uses “green” mesoscale photonics instead of electronic ones will come one step closer to practical realization, but the story has not ended.

## Data Availability

The data that support the findings of this study are available from the corresponding author upon reasonable request.
